# Exogenous insulin autoimmune syndrome: a case report and literature review

**DOI:** 10.3389/fendo.2025.1700742

**Published:** 2026-01-09

**Authors:** Yingyi Gao, Nan Wang, Zhishan Huang, Ning Bai

**Affiliations:** 1 Wuxi School of Medicine, Jiangnan University, Wuxi, Jiangsu, China; 2 Department of Endocrinology, Affiliated Hospital of Jiangnan University, Wuxi, Jiangsu, China

**Keywords:** exogenous insulin, HLA-DR4, hyperinsulinemic hypoglycemia, insulin autoantibody, insulin autoimmune syndrome

## Abstract

Exogenous Insulin Autoimmune Syndrome (EIAS) is a rare hypersensitivity reaction triggered by the administration of exogenous insulin. This reaction induces the production of insulin autoantibodies, leading to various clinical manifestations such as insulin resistance, hyperglycemia, recurrent hypoglycemic episodes, and allergic reactions. Due to limited clinical awareness, EIAS is often misdiagnosed or overlooked. Herein, we present a case of EIAS in a patient with type 2 diabetes mellitus, providing a detailed analysis of its clinical features, laboratory findings, differential diagnoses, therapeutic approaches, and clinical outcomes, supplemented by a literature review. This report aims to enhance clinicians’ diagnostic and therapeutic proficiency in managing this condition.

## 1 Introduction

Exogenous Insulin Autoimmune Syndrome (EIAS) is a rare disorder triggered by the administration of exogenous insulin, leading to an autoimmune response. It is characterized by the production of high titers of insulin autoantibodies (IAA), which reversibly bind to insulin. The dissociation of insulin from these immune complexes can cause unpredictable and severe glycemic variability, including recurrent hypoglycemic episodes and intermittent hyperglycemia. EIAS predominantly occurs in individuals with diabetes undergoing insulin therapy, particularly associated with specific insulin formulations and genetic predispositions (e.g., certain HLA genotypes) (1, 2).Diagnosis relies on the detection of insulin autoantibodies and observation of characteristic blood glucose fluctuations, requiring differentiation from other conditions such as insulinoma or factitious hypoglycemia. Management may involve switching insulin types, using corticosteroids or immunosuppressants, and implementing personalized glucose monitoring and control strategies.

## 2 Case presentation

A 58-year-old male was admitted to our department with complaints of “polydipsia and polyuria for 20 years, worsened by limb numbness for 1 month.” He was diagnosed with type 2 diabetes mellitus (T2DM) 20 years ago and initially managed with metformin and gliclazide. Due to suboptimal glycemic control, insulin degludec/insulin aspart (13 U in the morning, 17 U in the evening) was initiated five years ago. However, the patient continued to experience glycemic fluctuations along with episodic palpitations and sweating, which were relieved by food intake. Over the past month, he developed numbness and tingling in his hands and feet, prompting hospitalization for further management.

Past Medical History: No history of thiol-drug exposure or autoimmune diseases.

Family History: The patient had no past medical history of thiol-drug exposure or autoimmune diseases. The patient’s older brother also has diabetes.

### 2.1 Clinical assessment

On admission, the patient’s vital signs were as follows: temperature 36.4 °C, blood pressure 140/86 mmHg, heart rate 80 bpm, respiratory rate 14 breaths/min, and body mass index (BMI) 21.8 kg/m². The patient was alert and oriented, with clear speech. Cardiorespiratory auscultation revealed no significant abnormalities. No pitting edema was present in either lower extremity, and dorsalis pedis pulses were palpable bilaterally with normal characteristics. Neurological examination demonstrated diminished vibration sense and temperature perception in both lower limbs. Laboratory investigations showed normal liver and kidney function tests, as well as a normal lipid profile. Hemoglobin A1C (HbA1c) was 7.60%. Insulin autoantibody (IAA) level was markedly elevated at 34.92 U/mL (reference: 0–1), while glutamic acid decarboxylase (GAD) and islet cell antibodies (ICA) were negative. Fasting insulin was >300 μU/mL, and C-peptide was significantly below the lower limit of normal. HLA-DR4 was positive on PCR testing. Additional investigations included electromyography (EMG), which demonstrated mild polyneuropathy; ankle–brachial index (ABI) measurements of 0.94 (left) and 0.93 (right); and bone densitometry, which revealed reduced bone mineral content.

### 2.2 Diagnoses

1. Type 2 Diabetes Mellitus (T2DM)
2. Diabetic Peripheral Neuropathy
3. Exogenous Insulin Antibody Syndrome (EIAS)

### 2.3 Treatment course

**Figure 1:**
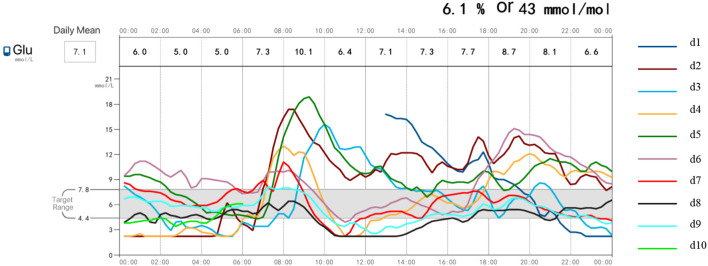
Continuous glucose monitoring (CGM) profile of the patient.

During the patient’s hospitalization, continuous glucose monitoring (**Figure 1**) revealed nocturnal blood glucose levels below 3.9 mmol/L, with no significant symptoms of hypoglycemia. After admission, the hypoglycemic regimen was adjusted by gradually reducing the insulin dose and adding acarbose 50 mg three times daily (TID) and dapagliflozin 5 mg once daily (QD), which resulted in a stable blood glucose level. At discharge, the regimen consisted of acarbose 50 mg TID, dapagliflozin 5 mg QD, and metformin 0.5g twice daily, achieving sustained glycemic control. Follow-up at 1 month showed an IAA level of 33.30 U/mL, decreasing to 18.09 U/mL at 3 months and 9.86 U/mL at 6 months, with no hypoglycemic episodes reported. Insulin levels also declined significantly (**Table 1**). During the treatment of diabetic patients, the primary goal is to ensure the absence of hypoglycemia, followed by achieving comprehensive glycemic control targets. Follow-up after the patient’s discharge pleased us as no further hypoglycemic episodes occurred; however, this was accompanied by a failure to reach the ideal glycated hemoglobin (HbA1c) target. The reasons are analyzed as follows:

**Table 1:** Changes in pancreatic islet function parameters in patients.

Timepoints	PG/mmol·L^-1^	INS/μU·mL^-1^	C-Peptide/ng·mL^-1^	IAA	HbA1c (%)
Fasting	7.04	144.25	0.97	34.92	7.6
2h postprandial	14.84	>300 11.89(PEG)	2.06		
Discharge (fasting)	5.6	113.28			
1 month post-discharge (fasting)	9.5				
1 month post-discharge (2hPP)		32.04	1.96	33.30	
3 months (fasting)	6.3	7.57		18.09	8.0%
3 months post-discharge (2hPP)	10.0	17.6			
6 months (fasting)	6.7	6.90		9.86	8.9%
6 months post-discharge (2hPP)	10.9	21.09	1.86

1. The patient experienced frequent hypoglycemic episodes prior to admission. Since HbA1c reflects the average blood glucose level over the preceding 2–3 months, the fluctuations between high and low blood glucose levels resulted in the HbA1c at admission not being significantly elevated.
2. The patient maintained better dietary and exercise management during hospitalization. After discharge, lifestyle changes led to less strict glycemic management compared to the inpatient period, resulting in a slight increase in HbA1c.
3. Concerned about the risk of recurrent hypoglycemia, the patient independently and appropriately increased snack intake, which may have contributed to the failure to achieve the target HbA1c.

In subsequent treatment, adjustments to the glucose-lowering regimen, psychological education for the patient, and repeated dietary guidance will be implemented to help the patient achieve further improvements in glycemic control.

**Figure 2:**
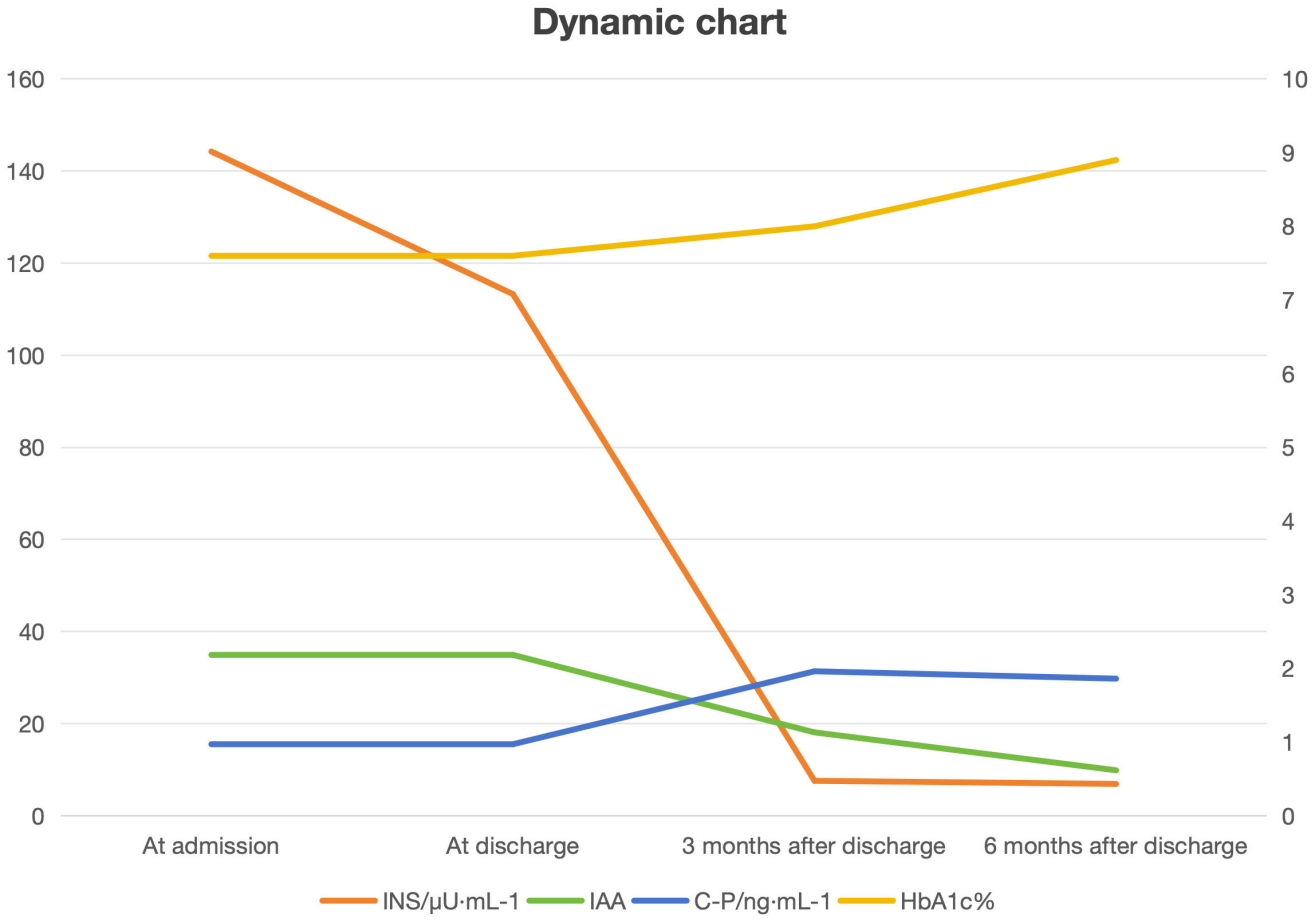
Dynamic chart.

The follow-up results of patients from admission to six months after discharge were compiled into trend charts. These charts visually demonstrate a progressive decline in IAA levels following scientific treatment of EIAS. The insulin indicators decreased rapidly after discharge and medication adjustment, and remained within the normal range during long-term follow-up. The patient, with a long history of diabetes and severe islet function impairment, showed no significant trend of change in C-peptide levels. (**Figure 2** Dynamic chart).

## 3 Discussion

### 3.1 Definition of EIAS

EIAS is a condition characterized by a clear history of exogenous insulin administration, in the absence of exposure to sulfhydryl-containing drugs or underlying autoimmune diseases, and with exclusion of insulinoma, type B insulin resistance, and other causes of hypoglycemia.

Its clinical characteristics include recurrent severe spontaneous hypoglycemia, elevated immunoreactive insulin levels, and high-titer insulin autoantibodies. This condition, along with type B insulin resistance and other hypoglycemic disorders, typically presents with frequent episodes of profound spontaneous hypoglycemia, excessively high immunoreactive insulin levels, and significant titers of insulin-binding autoantibodies (3, 4). Between 1970 and 2020, 120 cases of EIAS syndrome have been reported worldwide, with a female predominance (73 cases) compared to males (33 cases), primarily affecting patients around 60 years of age. Studies indicate that approximately 90% of EIAS patients had type 2 diabetes, while only about 5% had type 1 diabetes. EIAS also exhibits notable ethnic disparities, with disproportionately higher rates reported in Asian populations. Japan has the highest prevalence, followed by other East Asian countries such as Korea, whereas China has a relatively lower incidence. Meanwhile, cases are rare among Caucasians (5).Reported literature and genetic susceptibility studies suggest that EIAS may be associated with HLA-DR4, HLA-B15, and HLA-DR7 variants in Asian populations. A study by Y. Uchigata et al. found that IAS is more common in Asian populations and shows a strong association with HLA class II. This distribution is linked to alleles such as HLA-DR4, -B15, and -DR7 in these populations. Most typical Japanese patients tested positive for the Cw4/Bw62/DR4 haplotype, with DR4 demonstrating the strongest association (6–9). For instance, our patient tested positive for HLA-DR4 DNA using PCR, indicating a potential genetic predisposition to EIAS.

### 3.2 Diagnosis of EIAS

Currently, there is no definitive consensus on the diagnostic criteria for EIAS. Based on a combination of domestic and international literature on Insulin Autoimmune Syndrome (IAS), the following five points might serve as provisional diagnostic criteria for EIAS:

1. Recurrent spontaneous hypoglycemia with significant glycemic variability.
2. Documented history of exogenous insulin administration.
3. Positive IAA targeting exogenous insulin, typically showing IAA (+), GADA (-), ICA (-), and IA-2A (-).
4. High immunoreactive insulin levels with weak biological activity, demonstrating marked dissociation from C-peptide levels.
5. Absence of thiol-containing drug exposure and autoimmune diseases, with exclusion of insulinoma, type B insulin resistance syndrome, and other hypoglycemic etiologies (3, 4, 10).

Additional diagnostic tools include testing for free insulin and performing genetic analysis (11). For instance, the presented case showed glycemic variability with alternating hypoglycemia and hyperglycemia, positive IAA, insulin-C-peptide dissociation, and HLA-DR4 susceptibility, fulfilling the full EIAS diagnostic criteria (1, 9).

### 3.3 Pathogenesis of EIAS

Extensive literature suggests the following mechanism for EIAS: Under normal conditions, insulin binding to its receptors is crucial for maintaining glucose homeostasis. Exogenous insulin can act as an antigen, interacting with endogenous IAA through specific chemical and physical processes to form antigen-antibody complexes that impair insulin function. When conditions change (including pH, temperature, and ionic strength) or after postprandial hyperglycemia, these complexes destabilize and dissociate, releasing free insulin and IAA. This sudden surge of bioactive insulin, in turn, triggers hypoglycemia. Following meals, food intake stimulates endogenous insulin secretion; however, high antibody levels bind to insulin, reducing its bioactivity and leading to postprandial hyperglycemia. This hyperglycemic state further stimulates additional insulin secretion. As blood glucose decreases after meals, insulin-IAA complexes dissociate, releasing large amounts of active insulin and predisposing patients to hypoglycemia. Together, this cyclical process underlies the hallmark symptoms of EIAS (6, 11, 12).

## 4 Literature review

### 4.1 General information

Through documentation of this T2DM case presenting with concomitant EIAS, we conducted searches in multiple Chinese and English databases using relevant keywords and retrieval formulas, incorporating EIAS-related case reports from the past decade. Here, we summarized the distribution, clinical characteristics, basic information, laboratory findings, treatment methods, and outcomes of EIAS patients reported in previously reported cases. First, we searched articles from the past 10 years in databases including CNKI, Wanfang, PubMed, and Web of Science, ultimately selecting 30 eligible articles on EIAS (20 in Chinese and 10 in English), involving a total of 64 patients from three countries, predominantly from Asia. Among them, 59 cases were from China, 3 from Japan, and 2 from the United States (**Table 2**).

**Table 2:** General information.

Country	Studies (n)	Cases (n)	Proportion (%)
China	27	59	92.19
Japan	2	3	4.69
America	1	2	3.12
Total	30	64	100

### 4.2 Sex distribution

Among the 64 included patients, all reported their sex, with 42 being male and 22 being female. As shown in **Tables 3**, **2**, there was a male predominance (65.63%) compared with females (34.37%) (**Table 3**).

**Table 3:** Sex distribution.

Sex	Cases (n)	Proportion (%)
Male	42	65.63
Female	22	34.37
Total	64	100

### 4.3 Age distribution

Age analysis of EIAS patients showed broad variability (27–90 years), with the condition predominantly occurring in middle-aged and elderly populations: 19 cases were aged 30–59 years, and 42 cases were 60–89 years, as shown in **Table 4**.

**Table 4:** Age distribution.

Age at diagnosis (years)	Cases (n)	Proportion (%)
0-29	1	1.56
30-59	19	29.69
60-89	42	65.63
≥ 90	1	1.56
Unknown	1	1.56
Total	64	100

### 4.4 Insulin antibodies

The expression profiles of diabetes-related autoimmune antibodies in 64 patients diagnosed with EIAS, summarized according to the diagnostic criteria, are presented in **Table 5**.

**Table 5:** Insulin antibodies.

Insulin antibodies	Cases (n)	Proportion (%)
IAA (+)	64	100
GADA (+)	7	10.94
ICA (+)	4	6.25

### 4.5 Diabetes classification, disease duration, and insulin usage duration

All 64 cases clearly documented the diabetes classification of patients, with T2DM accounting for the majority. Among them, 60 cases reported the duration of diabetes, showing that most EIAS patients had a disease course exceeding five years. Of the 54 cases that documented insulin usage duration, although some patients had used insulin for more than five years, most clinical symptoms developed within one to four years after switching insulin types. Diabetes classification, disease duration, and insulin exposure duration were significantly correlated with EIAS occurrence. Patients with type 1 diabetes and special-type diabetes generally had a younger age of onset, longer disease duration, and extended insulin usage, resulting in higher EIAS incidence rates. Further details are presented in **Table 6**.

**Table 6:** Diabetes classification, disease duration, and insulin usage duration.

Diabetes classification	Cases (n)	Proportion (%)
T2DM	50	78.13
T1DM	9	14.06
LADA	3	4.69
Special Type Diabetes	1	1.56
IGT	1	1.56
Diabetes classification (years)		
< 1	8	12.50
1-4	9	14.06
5-10	17	26.56
≥ 10	26	40.63
Unknown	4	6.25
Insulin usage duration (years)		
< 1	17	26.56
1-4	19	29.69
5-10	9	14.06
≥ 10	9	14.06
Unknown	10	15.63
Total	64	100

### 4.6 Body mass index

A total of 51 Asian patients provided BMI data at diagnosis, including 50 Chinese patients and 1 Japanese patient. Recorded BMI values ranged from 13.2 kg/m² to 33.3 kg/m². According to classification standards, there were two underweight cases and 33 normal-weight cases. Notably, most EIAS patients had BMI values within the normal range, including the patient in the present case (21.8 kg/m²) (**Table 7**).

**Table 7:** Body mass index.

BMI(kg/m^2^)	Cases (n)	Proportion (%)
Underweight (< 18.5)	2	3.13
Normal range (18.5-24.9)	33	51.56
Overweight (25.0-29.9)	11	17.19
Obesity (≥ 30.0)	5	7.81
Unknown	13	20.31
Total	64	100

### 4.7 Types of insulin

A literature review of 63 EIAS patients analyzed the types of insulin used, which were categorized into five groups, including recombinant human insulin, synthetic insulin analogs, premixed formulations, premixed analog combinations, and dual-action analog preparations. Among these, insulin analogs accounted for the highest proportion, with rapid-acting insulin analogs being predominant, and long-acting insulin analogs mainly represented by insulin glargine. The second most common type was premixed insulin, with NovoMix 30 being the most frequently used across all insulin types (**Table 8**).

**Table 8:** Types of insulin.

Types of insulin	Classification of Insulin	Cases (n)
Recombinant Human Insulin		14
Short-acting	Novolin R	3
	Humulin R	3
	Gan & Shu Lin R	1
Intermediate-acting	Humulin N	3
	Novolin N	4
Insulin Analogs		41
Rapid-acting	NovoRapid/Aspart	16
	Humalog/Lispro	8
	Apidra/Glulisline	1
Long-acting	Lantus/Basalin	12
	Levemir	3
Ultra-long-acting	Tresiba	1
Premixed Insulin		39
Animal-derived premix	Wan Su Lin 30R	1
Human premix	Novolin 30R	6
	Humulin 70/30	3
	Gan & Shu Lin 30R	1
	Gan & Shu Lin 50R	1
	Humalog Mix 25R	2
Premixed analogs	NovoMix 30	22
	NovoMix 50	1
	Humalog Mix 25	1
	Humalog Mix 50	1
Dual insulin analog	Ryzodeg	3

### 4.8 Clinical manifestations

Based on statistical analysis, the clinical symptoms and blood glucose fluctuation patterns in EIAS patients can be broadly categorized into the following groups: hypoglycemia, hyperglycemia, alternating hyperglycemia-hypoglycemia, allergic reactions, ketoacidosis, autonomic nervous system symptoms, central nervous system dysfunction, and non-hypoglycemia.

Recurrent hypoglycemia was the most common manifestation, with nocturnal and early-morning episodes being the most frequent. Hyperglycemia was the primary clinical feature in 21 patients, with 18 cases reporting postprandial hyperglycemia as the dominant pattern. Allergic reactions (e.g., pruritus, urticaria, rash) or localized soft tissue reactions (e.g., injection-site induration, lipoatrophy) occurred in 11 patients. Lipoatrophy, a complication of long-term subcutaneous insulin administration, can lead to insulin accumulation at the atrophic site and disruption of normal insulin action rhythms, contributing to both hypoglycemia and postprandial hyperglycemia in EIAS patients.^(10)^

Ketoacidosis was reported in six cases, with a higher incidence in patients with T1DM and specific diabetes subtypes. Autonomic symptoms (e.g., sweating, palpitations) and central nervous system dysfunction (e.g., syncope, coma) were observed in severe cases. Notably, three patients showed no obvious hypoglycemic symptoms, which is significant given the diagnostic importance of hypoglycemia in EIAS (**Table 9**).

**Table 9:** Treatment approaches and symptom remission duration.

Clinical manifestations	Specific Clinical Presentations	Cases (n)
Hypoglycemia	Nocturnal/Early morning	26
	Fasting	21
	Postprandial	3
	Exercise-induced	1
Absence of hypoglycemia		3
Hyperglycemia	Daytime	3
	Postprandial	18
Glycemic variability		17
Allergic reactions (rash, localized soft tissue reactions)		11
Ketoacidosis		6
Hypoglycemia with Autonomic nervous system symptoms		5
Hypoglycemia with Central nervous system dysfunction		7

### 4.9 Treatment approaches and symptom remission duration

Among 64 cases, 50 patients were switched to oral antidiabetic agents, including α-glucosidase inhibitors (acarbose and voglibose), metformin, and DPP-4 inhibitors (gliptins), with α-glucosidase inhibitors being the most frequently prescribed. Twenty patients had adjustments in insulin type or injection method/site. In addition, 25 patients achieved symptom relief through dietary and lifestyle modifications combined with oral medications, including three cases managed by lifestyle changes alone. For T1DM-associated EIAS, insulin therapy remains essential due to the limited efficacy of oral agents. These cases often require concurrent glucocorticoids and antihistamines to manage allergic reactions.

Follow-up data from 41 cases showed that 22 patients experienced cessation of hypoglycemia within one to five months after treatment adjustment, while six patients achieved complete remission within one month. Regarding IAA seroconversion, nine cases documented IAA turning negative, although most studies did not report precise timelines. Symptomatic improvement occurred within two weeks in nearly all patients, accompanied by stable glycemic control. Among patients followed for more than one year, over 50% achieved IAA negativity (**Table 10**).

**Table 10:** Treatment approaches and symptom remission duration.

Treatment approaches	Specific medications	Cases (n)
Dietary and lifestyle modifications		25
Insulin regimen adjustments		20
Insulin discontinuation with oral antidiabetic agents	α-Glucosidase inhibitors	41
	Biguanides	19
	DPP-4 inhibitors	16
	Non-sulfonylurea insulin secretagogues	6
	GLP-1 receptor agonists	3
	Thiazolidinediones	3
	Sulfonylureas	1
Glucocorticoid therapy		11
Immunotherapy	Mycophenolate mofetil	3
	Rituximab	1
Plasmapheresis		1
Anti-allergy treatment		3
Time (months)	Stable blood glucose, symptomatic improvement	IAA negative
≤ 1	6	2
1-5	22	1
6-12	7	3
≥ 12	6	3

In summary, based on our hospital’s management of a typical EIAS case with clinical manifestations consistent with the diagnosis, insulin was promptly discontinued, and the glucose-lowering regimen was adjusted to include metformin, acarbose, and dapagliflozin. Dapagliflozin, a sodium-glucose cotransporter-2 (SGLT-2) inhibitor, lowers blood glucose by inhibiting renal glucose reabsorption and promoting urinary glucose excretion. Its insulin-independent mechanism reduces glycemic variability and the risk of hypoglycemia, while providing cardiovascular and renal protection. Although previous literature has not reported the use of these medications in EIAS, this patient achieved good glycemic control with SGLT-2 inhibitors, though ketone monitoring remains essential to prevent ketoacidosis. For EIAS patients with type 2 diabetes and higher BMI, discontinuing insulin and switching to GLP-1 receptor agonists is recommended. These agents lower glucose through glucose-dependent insulin secretion, glucagon suppression, delayed gastric emptying, and appetite reduction, significantly decreasing the risk of hypoglycemia (13).In addition, dapagliflozin may be added to support weight loss and blood pressure control.

Clinicians should diagnose EIAS based on a history of hypoglycemia, exogenous insulin use, diabetes autoantibodies, and elaboratory results. Detecting diabetes autoantibodies improves diagnostic accuracy and helps prevent severe outcomes from misdiagnosis. Even IAA-negative cases cannot exclude EIAS, necessitating follow-up or alternative testing. Some EIAS patients may lack hypoglycemic episodes, leading to underdiagnosis or misclassification as brittle diabetes. For these patients, a comprehensive evaluation is essential to prevent complications.

## Data Availability

The original contributions presented in the study are included in the article/supplementary material. Further inquiries can be directed to the corresponding author.
